# Important Mycosis of Wildlife: Emphasis on Etiology, Epidemiology, Diagnosis, and Pathology—A Review: PART 2

**DOI:** 10.3390/ani12151897

**Published:** 2022-07-26

**Authors:** Iniobong Chukwuebuka Ikenna Ugochukwu, Iasmina Luca, Nuhu Abdulazeez Sani, Jacinta Ngozi Omeke, Madubuike Umunna Anyanwu, Amienwanlen Eugene Odigie, Remigius Ibe Onoja, Ohiemi Benjamin Ocheja, Miracle Oluchukwu Ugochukwu, Olabisi Aminah Makanju, Chioma Inyang Aneke

**Affiliations:** 1 Department of Veterinary Pathology and Microbiology, University of Nigeria, Nsukka 410001, Nigeria; iniobong.ugochukwu@unn.edu.ng (I.C.I.U.); jacinta.omeke@unn.edu.ng (J.N.O.); madubuike.anyanwu@unn.edu.ng (M.U.A.); remigius.onoja@unn.edu.ng (R.I.O.); chioma.aneke@unn.edu.ng (C.I.A.); 2 Department of Veterinary Medicine, University of Bari, 70010 Bari, Italy; amienwanlen.odigie@uniba.it; 3 Department of Pathological Anatomy and Forensic Medicine, Faculty of Veterinary Medicine, Banat’s University of Agricultural Sciences and Veterinary Medicine, 300645 Timișoara, Romania; 4 Department of Veterinary Pathology, University of Abuja, Abuja 900109, Nigeria; nuhu.sani@uniabuja.edu.ng; 5 Department of Veterinary Public Health and Preventive Medicine, University of Benin, Benin City 300238, Nigeria; 6 Department of Biosciences, Biotechnologies and Biopharmaceuticals, University of Bari, 70010 Bari, Italy; ohiemi.ocheja@uniben.edu; 7 Department of Veterinary Physiology and Biochemistry, University of Benin, Benin City 300238, Nigeria; 8 University of Nigeria Medical Centre, Nsukka 410001, Nigeria; miramimiugochux@gmail.com; 9 National Veterinary Research Institute, Vom 930010, Nigeria; olabisi.makanju@nvri.gov.ng

**Keywords:** wildlife, fungal diseases, zoonosis, diagnosis, pathology

## Abstract

**Simple Summary:**

The conservation of wildlife species is a major goal globally, due to the declining number of total populations. Fungal diseases are frequently diagnosed in veterinary practice, so knowing their characteristics, along with knowing the appropriate laboratory methods to make a correct diagnosis, are extremely important for the veterinarian. This article is the continuation of an extensive review that presents the main fungal diseases of wild animals. In this part, the second, four of these mycoses are discussed (dermatophytosis, coccidioidomycosis, blastomycosis, and sporotrichosis).

**Abstract:**

Wild animals are an important component of the ecosystem, and play a major role in it. However, in recent years, there has been an astronomical increase in the incidence of wildlife mycotic diseases leading to wildlife extermination. It is important to note that most of these mycotic diseases are zoonotic, and since there is a lot of attention given to zoonosis of a bacterial or viral origin in recent times, it is important to look into the mycotic diseases which may have zoonotic potential. Previously, the authors expatiated on some major wildlife mycotic diseases. In this review, we shed light on the etiology, epidemiology, diagnosis, pathogenesis, pathogenicity, macroscopic and microscopic pathology, and hematological and serum biochemical findings of dermatophytosis, coccidioidomycosis, blastomycosis, and sporotrichosis, which are very important mycoses of wildlife.

## 1. Introduction

Fungi are not yet considered a major public health issue, whereas diseases caused by bacteria and viruses have been identified as a more critical public health concern for many decades [1,2]. 

Opportunistic fungi are found in various natural habitats, and can only occasionally produce infection after penetrating the intact skin of a host with a feeble immune system, or in cases of other devitalizing conditions [3]. Lamentably, some fungal diseases with zoonotic potential have been ignored; thus, the strategies for controlling their spread remain insufficient [4]. From a universal perspective, zoonotic infections have been identified for many centuries. These are part of the majority of emerging and re-emerging infectious diseases that have impacted the global economy substantially [5]. Infectious diseases emerge through diverse modes. The same applies to zoonotic diseases; hence, the dynamic interplay between wildlife, domestic animals, and humans is important to maintain their zoonotic potential. The viral and bacterial zoonotic diseases with a wildlife origin have taken center stage with the reports of infectious pandemics; on that account, it is important to emphasize wildlife mycoses which could act as a source of zoonotic risks [6,7,8].

The early diagnosis of fungal infection is essential to effective treatment [9]. Therefore, the laboratory diagnosis of mycotic infections includes direct microscopic examination; histopathology; microbial culture; antigen detection, such as in the use of immunohistochemistry; serology-immunodiffusion (ID); complement fixation (CF); enzyme immunoassay (EIA); and molecular tests, such as in situ hybridization and polymerase chain reaction (PCR). Imaging techniques, such as magnetic resonance imaging (MRI) and laser microdissection, have also been helpful in the diagnosis of fungal diseases [9,10,11].

Fungi are a relevant cause of diseases in wild animals. They cause mycosis, and allergic disease involving the development of hypersensitivity or mycotoxicosis [12]. Inhalation is the primary route for the transmission of most fungi-causing mycosis [12]. In this review, as a continuation, we highlighted the etiology, transmission, epidemiology, diagnosis, pathology, and impact of some other important mycoses of wildlife, such as dermatophytosis, coccidioidomycosis, blastomycosis, and sporotrichosis.

## 2. Dermatophytosis

This mycotic disease, also known as tinea corporis, capitis tinea, or ringworm, is a very contagious and infectious skin disease [13,14,15]. Dermatophytoses are superficial infections of the skin, stratum corneum, nail beds, and hair follicles of humans and animals [14,16,17]. Of the three dermatophytic genera recognized, *Epidermophyton* exclusively affects humans, whereas *Microsporum* and *Trichophyton* mainly affect animals [18]. Dermatophytosis is an important public health problem worldwide [14,19]. It is the most frequent dermatologic problem confronting veterinarians because it affects a wide range of domestic and wild animals [14,20]. Dermatophytes are involved in various fungal infections that dermatologists and veterinarians are called upon to treat [18]. Dermatophytosis is characterized by localized or generalized alopecic lesions with erythema, usually non-pruriginous, being of dominant significance in veterinary medicine due to its swift dissemination [21].

### 2.1. Etiology

The genera, *Trichophyton*, *Microsporum*, and *Epidermophyton*, together make up the fungal agents that cause dermatophytosis [17]. *Trichophyton gallinae* is the prime cause of ringworm or fowl favus in birds [12]. It has been isolated from poultry birds, several species of wild birds, companion animals, humans, and other mammals [12]. *Trichophyton erinacei* is the most diagnosed in European hedgehogs (*Erinaceus europaeus*) [22] and African pygmy hedgehogs (*Atelerix albiventris*) [23]. In the USA, among three infected cougars (*Puma concolor*), *Microsporum gypseum* was isolated from one, whereas *Trichophyton mentagrophytes* were recovered from the remaining two [24,25]. *M. canis* caused a small focus of ringworm in an African lion (*Panthera leo*) [25]. *Microsporum* spp. were also isolated from the haircoat of a tiger (*Panthera tigris*) [25]. In Brazil, an ocelot (*Leopardus pardalis*), a lion, and a tiger presented lesions likened to dermatophytosis, but *Microsporum gypseum* was only isolated from the ocelot [25]. A direct wet mount of skin scrapings from impalas (*Aepyceros melampus*) had ectothrix spores (fungal conidia arranged outside the hair shaft) and yielded *Microsporum gypseum* on culture [26]. A gazelle (*Gazella gazella*) had a *Microsporum gypseum* infection [26], and two others were reported to have a concurrent infection with *Trichophyton schoenleinii* and *Microsporum gypseum*. The disease has spread and has been identified in giraffes (*Giraffa camelopardalis*), with *Trichophyton schoenlenii* being isolated on culture [26]. *Trichophyton tonsurans* has been isolated from a brown fish owl (*Ketupa zeylonensis*) [27], *Trichophyton concentricum* from an Asian elephant (*Elephas maximus*) [27], *Microsporum nanum* from a leopard (*Panthera pardus*), *Epidermophyton floccosum* from a white tiger (*Panthera tigris*) [27], *Microsporum* *audouinii* from a chimpanzee (*Pan troglodytes*), and *Microsporum canis* from a maned wolf (*Chrysocyon brachyurus*) [21].

### 2.2. Epidemiology

The predisposing factors to dermatophytosis include a compromise in the integrity of the mucosa or skin surface, exposure to ultraviolet radiation, physical-chemical factors, the presence of concurrent disease, or the effect of immunosuppressing medications [25,28,29]. Geographical location, season, and living conditions affect the prevalence of dermatophytosis [30]. Generally, countries with a hot and humid climate have a higher prevalence of dermatophytosis [25,31]. Ringworm in wild birds is highly contagious and is transmitted by direct contact or contact with a contaminated environment. In infected scales or skin lesions that slough from the body, the fungus (dermatophyte) can remain viable at room temperature for up to one year [12].

### 2.3. Diagnosis

A direct physical examination of hair and scales is a field technique used to confirm the presence of dermatophytosis [32,33]. Dermoscopy is a non-invasive diagnostic technique used in medical practice that allows for illuminated magnification of the skin, but is also used in veterinary practice for the diagnosis of dermatophytosis [32,33]. Fungal culture, Wood’s lamp examinations, or PCR have been used to monitor fungal infections. The histological examination of biopsy tissue is rarely used in the diagnosis of dermatophytosis [32], but histochemistry with periodic acid–Schiff (PAS), Grocott’s methenamine silver, and calcofluorwhite (CFW) stains has been helpful in the diagnosis of dermatophytes [34]. In practice, Sabouraud dextrose agar [23], potato dextrose agar, and malt extract agar, as reported by Le Barzic et al. (2021), can be used to isolate and identify certain species of *Trichophyton* [22]. A multiplex PCR which can detect *Trichophyton* spp., *Microsporum canis*, and *M. audouinii* directly in clinical specimens has been developed. Random amplified polymorphic DNA, (RAPD)-PCR, has been used to differentiate *M. canis* isolates from different animal species, including wild foxes (*Vulpes vulpes*) [35,36]. Uniplex PCR-ELISA has also been utilized in the identification of *M. canis* [35].

### 2.4. Pathology

#### 2.4.1. Pathogenicity and Pathogenesis

The ability to adhere and adapt to the host environment is critical for the establishment of infection by a dermatophyte. In the early stages of fungal–host interaction, transcription factors, such as PacC, Hfs1, and heat shock proteins, are involved in sensing and adapting to the acidic pH of the skin [17]. During dermatophyte growth, the extracellular pH shifts from acidic to alkaline, creating an environment for optimal activity of most of the known keratinolytic proteases [17]. Certain species of *Trichophyton* can survive for up to one year on the skin of affected animals [37].

#### 2.4.2. Clinical Signs

Dermatophytosis may appear as one or as a combination of hair loss, papules, scales, crusts, erythema, hyperpigmentation, and pruritus [32]. In wild red foxes (*Vulpes vulpes*), *Trichophyton mentagrophytes* associated with dermatophytosis appeared as diffused alopecic skin areas and scattered crusty *foci* [16]. Dermatophytic lesions occurred mainly in the abdominal, thoracic, and hindlimb regions in an Asian elephant (*Elephas maximus*) with a mixed-dermatophyte infection [27]. During the infection, the elephant did manifest inappetence, but was dull and pruritic. In an infected leopard (*Panthera pardus*), aside from pruritus, depression, and anorexia, dermatophytosis manifested as scab formation and alopecia in the thoracic, dorsum, and lumbar regions, as well as the distal part of the tail [27]. In three brown fish owls (*Ketupa zeylonensis*), crusted granular type lesions with defoliating scales were observed on the eyelid. Decreased flight, inappetence, and scab formation on the legs were also observed in these owls [27]. In European hedgehogs (*Erinaceus europaeus*), *Trichophyton erinacei* is most isolated from the head [22].

#### 2.4.3. Macroscopic Findings

Desquamation, alopecia, and erythema of the skin are the major gross lesions observed in dermatophytosis [25,38]. *Microsporum*-*audouinii*-associated dermatophytosis in a chimpanzee (*Pan troglodytes*) manifested as non-pruriginous alopecia, erythema, and scales [21]. In a maned wolf (*Chrysocyon brachyurus*), *Microsporum canis* infection appeared as alopecic, but non-pruritic, lesions with scales and crusts on the left pina region [39]. Alopecic loss of feathers on the head was observed in a loon (*Gavia* sp.) that had lesions that resembled ringworm [12].

#### 2.4.4. Histopathological Findings

In porcupines (*Erethizon dorsatum*), diffused severe epidermal hyperkeratosis; mild hyperplasia; and dermatitis with, predominantly, lymphocytes and plasma cells were reported [40]. Dermatophytes appeared as hyphae and spores in hair shafts, and follicular and epidermal keratin [40].

## 3. Coccidioidomycosis

Coccidioidomycosis, also known as Valley fever, California fever, or San Joaquin Valley fever, is a potentially disastrous mycotic disease, as its incidence is increasing [41,42,43]. Coccidioidomycosis presents in different forms, ranging from a mild, self-limiting febrile illness to serious and deadly infection [42,44].

### 3.1. Etiology

Coccidioidomycosis is caused by a soil-dwelling fungus of the genus *Coccidioides*, which consists of two species: *C. immitis* and *C. posadasii* [43,44,45].

### 3.2. Epidemiology

Infection by *Coccidioides* occurs following the inhalation of arthroconidia from both *Coccidioides species*, which are endemic to arid and semi-arid regions of North America [46,47]. *Coccidioides* species infect reptiles, birds, humans, and other mammals, including wild mammals [42,44]. The lifecycle and infection rates of *Coccidioides* are determined by climatic changes and environmental factors [44].

Geographically, coccidioidomycosis is an endemic mycosis, since the etiologic agent is found in soil in certain areas of the western hemisphere [48]. *C. immitis* exists as a mold, and grows as branching, septate hyphae bearing specialized conidia called arthroconidia. [48,49]. On maturation, arthroconidia are resilient and may remain viable for a long time until suitable environmental conditions emerge for their germination [48,50]. Although the usual habitat of *C. immitis* is soil, its hardy arthroconidia are easily airborne, and, thus, are inhaled by animals and humans who become accidental hosts [48].

### 3.3. Diagnosis

The direct examination by wet mount using 10% potassium hydroxide (KOH), the isolation of fungus by culture, and routine histopathologic examinations supported by special histochemical stains such as PAS and CFW fluorescent stain (which offers the best detection of the organism) have all proved important in the diagnosis of coccidioidomycosis [35]. Other confirmatory tests, such as immunohistochemistry, PCR, ELISA, and electron microscopy, have all been used in synergy for the definitive diagnosis of coccidioidomycosis [35,43].

### 3.4. Pathology

#### 3.4.1. Pathogenicity and Pathogenesis

Infection is triggered following the inhalation of arthroconidia, and once the spore is in the host’s respiratory system, it undergoes a thermally-induced switch to the pathogenic and parasitic phase of the fungus’ life cycle [51]. It is speculated that ammonia release is involved in the virulence of *Coccidioides* [52]. This fungus is sensitive to environmental pH changes [47,52]. For example, when either the saprophytic or parasitic phase is in acidic cultures, the fungus releases NH_3_/NH_4_^+^ and urease enzyme, which catalyzes the hydrolysis of urea into two ammonia molecules that neutralize the acid [47,52,53]. Ureases are well-characterized virulence factors in other microorganisms [47].

#### 3.4.2. Clinical Signs

The clinical signs shown by baboons (*Papio* sp.) and chimpanzees (*Pan troglodytes*) with coccidioidomycosis were majorly those of dyspnea, tachypnea, lethargy, or neurologic and locomotion abnormalities [43], whereas the clinical signs exhibited by an infected macaque (*Macaca sp.*) were mostly coughing, gagging, urinary incontinence, paresis of the hind limb, dyspnoea, ruptured bladder, diarrhea with some hemorrhagic dehydration, and subcutaneous edema [43].

#### 3.4.3. Macroscopic Findings

In sea lions (*Zalophus californianus*) with disseminated coccidioidomycosis, pleuritis, pleural effusion, peritonitis, peritoneal effusions, lymphadenopathy, myocarditis/epicarditis, and thickened pericardial sacs were observed [54]. In a harbor seal (*Phoca vitulina*), *Coccidioides* disseminated to the thoracic and abdominal lymph nodes, liver, and spleen, thereby triggering multifocal pulmonary granulomas and emphysema [54]. In baboons (*Papio* sp.), gross lesions were observed within the thoracic organs, including adhesions, granulomas, abscesses, or ulcers [43]. In some baboon carcasses, the lungs had dark-brown-to-black discoloration, with black-to-brown fluid seen throughout all lung lobes on the cut section, whereas in some other baboon carcasses, firm lungs that were mottled gray-red-pink with a production of suppurative exudate on sectioning were observed. There were also granulomas in the spleen and associated lymph nodes in these baboon carcasses [43]. In chimpanzees (*Pan troglodytes*), aside from granulomatous lesions in the respiratory system, abscesses, hydropericardium, and ascites containing fluid that was clear, seropurulent with fibrin tags or suppurative were noted [43]. In macaques (*Macaca* sp.), the lung and thoracic wall lesions had yellow *foci* of abscessation, nodules, miliary *foci*, or areas of infarction. Additionally, subcutaneous and scrotal edema, hydropericardium, and ascites were also present [43]. Grossly, multifocal-to-coalescing areas of consolidation in the right and left cranial lung lobes were observed during necropsy of the macaques [55].

#### 3.4.4. Histopathological Findings

In sea otters (*Enhydra lutris*), respiratory system involvement with pyogranulomatous pleuritis, pleural effusion, and pulmonary nodules was a common finding [54]. Pneumothorax was also detected, along with peritoneal effusion and peritonitis. All otters with sufficient data for determination had gross evidence of fungal hilar, mediastinal, peripheral, axillary, retropharyngeal, or inguinal lymphadenopathy [54]. The plaques were composed of pyogranulomatous inflammation with fungal spherules. Enlarged abdominal lymph nodes were also noticed. The spleen, meninges, brain, and liver were common sites for fungal dissemination. A thickened pericardial sac and fungal myocarditis or epicarditis were also observed. Coccidioidal ophthalmitis was also confirmed by histopathology [54]. In macaques (*Macaca* sp.), pulmonary consolidation with pyogranulomatous bronchopneumonia and the presence of round, non-budding fungal yeast structures that were morphologically consistent with *Coccidioides immitis* was observed [55].

## 4. Blastomycosis

### 4.1. Etiology

Blastomycosis is caused by Blastomyces dermatitidis (family *Ajellomycetaceae*). This fungus is a thermally dimorphic fungus, and causes diseases of lethal proportions in humans, canids (wolves (*Canis lupus*), coyotes (*Canis latrans*)), and other mammals [56]. Over time, the disease has been reported in aquatic mammals (sea lions (*Zalophus californianus*), dolphins (*Delphinus delphis*)), elands (*Taurotragus oryx*), ferrets (*Mustela furo*), Indian fruit bats (*Pteropus medius*), American black bears (*Ursus americanus*), African lions (*Panthera leo*) [57,58], and lemurs (*Lemuroidea*) [59]. It is not easily isolated from the soil, its environmental reservoir [60]. *B. gilchristii* is the other fungal etiologic agent that can cause blastomycosis [61].

### 4.2. Epidemiology

Blastomycosis is primarily a canine disease [56]. Infection usually occurs through the inhalation of airborne conidia liberated from the mold phase, often resident in moist, acidic, sandy soils enriched with decaying organic matter and animal droppings [56,60]. In Canada, red foxes (*Vulpes vulpes*) and wolves (*Canis lupus*) are the main reservoirs and main vectors involved in transmitting the disease [60]. The aerosolization of conidia is promoted by disturbances to the soil, such as excavation or soil burrowing [56,62]. The geographic range of endemicity for animal and human blastomycosis includes North America, where it primarily occurs in states and provinces along the Great Lakes, and Ohio, Mississippi, Missouri, and St. Lawrence rivers [56,62]. Autochthonous blastomycosis has also been reported in Africa [56].

### 4.3. Diagnosis

The standard technique for diagnosing blastomycosis is the isolation of *Blastomyces* spp. from a clinical specimen using Sabouraud’s dextrose or potato dextrose agar [56,62]. *Blastomyces* colonies are white-to-buff in color, and usually appear within 10–14 days of incubation at 25–30 °C. However, some strains may require up to 6 weeks of incubation to grow [56,62]. Microscopically, *B*. *dermatitidis*/*gilchristii* characteristically has conidiophores of varying lengths resembling “lollipops” [56,62]. A direct examination could also be performed using KOH followed by negative staining with a Diff-Quik staining kit [35]. *Blastomyces* is identified by its characteristics, which include a classic appearance of large, round, multinucleate yeast-like cells with thick and double refractile walls [56,63]. Thoracic radiography and *B. dermatitidis* agar gel immunodiffusion serology could be carried out in endemic areas to rule out blastomycosis [57]. Fluorescent antibody techniques have also been utilized in the diagnosis of blastomycosis [64]. Nested PCR and skin tests (blastomycin) have also been employed in the detection of *Blastomyces* [35].

### 4.4. Pathology

#### 4.4.1. Pathogenicity and Pathogenesis

Following the inhalation of mycelial fragments, conidia, and spores, *Blastomyces* spp. is converted into a pathogenic yeast (which is difficult to phagocytose due to the thick capsule) through a reversible morphological change [65]. This change enables the yeast to evade the host immune defensive mechanism, thereby causing pneumonia and disseminated disease [65]. In the yeast phase, *Blastomyces* regulates the essential virulence factor, BAD1, which facilitates adhesion to the host, impairs activation of immune cells, and blunts the cytokine release [65,66]. *Blastomyces* yeast also secretes dipeptidyl-peptidase IVA (DPPIVA) enzyme, which blunts the action of cytokines released from host immune cells [65,67]. Thus, blastomycosis presents clinical signs ranging from subclinical infection, acute pneumonia, chronic pneumonia mirroring the clinical signs of tuberculosis or malignancy, and acute respiratory distress syndrome [35,65].

#### 4.4.2. Clinical Signs

A sea lion (*Zalophus californianus*) with blastomycosis showed anorexia and lethargy, which resulted in emaciation and its eventual death [68]. Anorexia and dry inspiratory rales were also exhibited by an African lion (*Panthera leo*) [64]. Six wild felids (two Asian lions (*Panthera leo persica*), and one each of African lion (*Panthera leo*), Siberian tiger (*Panthera tigris altaica*), cheetah (*Acinonyx jubatus*), and snow leopard (*Panthera uncia*)) diagnosed with blastomycosis showed signs of lethargy, anorexia, cachexia, dyspnea, ataxia, and paresis [57].

#### 4.4.3. Macroscopic Findings

Postmortem examination of a wild coyote (*Canis latrans*) revealed randomly distributed white nodules in the lungs [69]. A necropsy examination of a sea lion (*Zalophus californianus*) diagnosed with blastomycosis revealed a focally perforated jejunum; abundant brown-to-black, fetid-smelling fluid in the abdominal cavity; discolored serosal surfaces of all abdominal organs; moderately enlarged mesenteric lymph nodes; and diffusely congested lungs with fibrosis of the right caudal lung lobe and a calcified nodule which contained purulent material centrally [68]. The lungs of wild red foxes (*Vulpes vulpes*) with blastomycosis consistently had nodules, and B. dermatitidis yeasts were recovered from them [60]. The lymph nodes and skin of these foxes were also affected, but not extensively [60]. In dead red foxes, B. dermatitidis was recovered from lungs with severe, multifocal-to-coalescing granulomatous pneumonia, whereas trapper-killed red foxes had small numbers of well-circumscribed pulmonary lesions [60]. Chronic granulomatous pulmonary mycosis has also been reported in ferrets (*Mustela furo*) that died of blastomycosis [70,71]. Skin, bone, and central nervous system lesions have also been described unspecifically [72].

#### 4.4.4. Histopathological Findings

The lung section of a wild coyote (*Canis latrans*) diagnosed with blastomycosis revealed multifocal coalescing granulomas with abundant macrophages, numerous neutrophils, fibroblasts, plasma cells, and lymphocytes [69]. Abundant intracellular and extracellular thick-walled, refractile, spherical yeasts (PAS-positive and broad-based single budding yeasts) were observed within these granulomas. The lung sections of sea lions (*Zalophus californianus*) with disseminated blastomycosis revealed extensive multifocal-to-coalescing aggregation of large numbers of macrophages and neutrophils, and scattered giant cells and lymphocytes admixed with fibrin and abundant necrotic debris [68]. Vascular thrombosis was also observed in these histologic sections. Several portions of the intestine (including perforated sites) contained aggregations of pyogranulomatous-to-granulomatous infiltrates with necrosis. Mild pyogranulomatous infiltration admixed with yeasts was observed within the spleen and liver. Moderate hepatic hemosiderosis, nodular and diffuse adrenal cortical hyperplasia, and nodular thyroid hyperplasia were also observed [68]. The lungs and tracheobronchial lymph nodes of four felids (tigers (*Panthera tigris*) and cheetahs (*Acinonyx jubatus*)) yielded *B. dermatitidis*, which triggered a florid pyogranulomatous reaction [57]. Pyogranulomatous encephalomyelitis was observed in the tiger, whereas in the cheetah, a single pulmonary granuloma was observable [57].

#### 4.4.5. Hematological and Serum Biochemical Findings

Six non-domestic felids diagnosed with blastomycosis showed variable and nonspecific hematological and serum biochemical pictures, which included leukocytosis, monocytosis, moderate left shift of neutrophilia, moderate hypercalcemia, hyperproteinemia, and hyperglobulinemia [57].

## 5. Sporotrichosis

Sporotrichosis is a chronic fungal disease of cutaneous and subcutaneous tissues [73], and is recognized as the most common subcutaneous mycosis [74]. It is an emerging zoonotic disease [74,75] caused by the dimorphous fungus, Sporothrix schenckii (*Ophiostomataceae* family), which has a worldwide distribution, especially in tropical and subtropical regions. Infection generally occurs following the inoculation of trauma by soil, plants, and organic matter contaminated with the fungus [73].

### 5.1. Etiology

For many years, *Sporothrix schenckii* has been considered the only etiologic agent of sporotrichosis. However, more *Sporothrix* species have been identified due to advances in research. S. brasiliensis and S. globosa were recently described, and they are reported to be prevalent in Latin America and East Asia, respectively [76,77]. These organisms, together with S. schenckii and S. luriei, make up the “pathogenic group” of the genus, Sporothrix [78]. S. mexicana also has a wide distribution [79]. There is variation in the virulence of *Sporothrix* species. *S*. *schenckii* causes benign chronic subcutaneous mycosis; *Sporothrix brasiliensis* is highly virulent, whereas *Sporothrix globosa* more often causes fixed cutaneous lesions [80].

### 5.2. Epidemiology

*Sporothrix schenckii* complex species thrive in warm and humid climates. Some may be thermo-resistant, whereas others grow at a pH ranging from 3.5 to 9.6 [73,81,82]. The sources of infection, and the transmission and distribution modes also differ between species [83]. The disease has been diagnosed in a wide variety of animal species, such as camels (*Camelus* sp.), guinea pigs (*Cavia porcellus*), foxes (*Vulpes vulpes*), dolphins (*Delphinus delphis*), armadillos (*Dasypus novemcinctus*), and non-human primates [84].

The major route of entry is percutaneous, through injury and wounds in contact with contaminated material. Aerogenous entry is not common, but entrance through this route incites pulmonary cases [73,81]. Sporotrichosis has a global distribution, but the disease is commonly seen in tropical and subtropical regions of the world [85]. Notably, zoonotic transmission in isolated cases and small outbreaks of sporotrichosis have been reported [73].

The fungus is sensitive to common disinfectants, especially alcohol-based ones [86].

### 5.3. Diagnosis

The gold standard for the diagnosis of sporotrichosis is the isolation of the organism from active lesions, pus, secretions, or biopsy [73,74,83]. However, ancillary techniques, including serological, histopathological, and molecular approaches, have been adopted in the diagnosis of sporotrichosis [73]. Direct mycological examination using KOH was used in the diagnosis of lymphocutaneous and cutaneous sporotrichosis, whereas Gram, Giemsa, PAS, and Grocott-Gomori’s staining (silver staining) have been useful in diagnosing disseminated sporotrichosis [87,88].

In felines, sporotrichosis is characterized by a high fungal load in the lesions; as such, a direct examination is used for the rapid diagnosis of the disease in this species [83,89]. In the direct examination, yeast cells that are round and often elongated (having a “cigar” shape) are observed [73]. Although indirect examination does not allow the differentiation of Sporothrix species, it is useful in ruling out other cutaneous sporotrichosis infections [83,90]. A cutaneous sporotrichin skin test detects delayed hypersensitivity, and could be used in epidemiological studies [90]. PCR and polymerase chain reaction–restriction fragment length polymorphism (PCR-RFLP) techniques are also used in the diagnosis of sporotrichosis [91,92,93].

### 5.4. Pathology

#### 5.4.1. Pathogenicity and Pathogenesis

There have been dissimilarities in the pathogenicity of organisms that make up the *S. schenckii* complex. For example, *S. brasiliensis* has been reported to be more virulent than *S. schenckii* [73,94], but both produce similar clinical descriptions [94]. The virulence of the *S*. *schenkii* complex is attributed to fungal dimorphism, thermotolerance, conidia production, extracellular proteins, epithelial adhesion, and the antigenic properties of l-rhamnose [73,94]. *S. schenckii*, *S. brasiliensis,* and *S. globosa* have the highest zoonotic potential [95].

#### 5.4.2. Clinical Signs

The main lesions noticed on the skin are consequences of the fistulated lymph nodes. Thus, ulcers, crusts, microabscesses, nodules, or verrucous lesions can be noticed [96].

Crustaceous skin alterations and alopecia were observed in an Eastern quoll (*Dasyurus viverrinus*) with S. humicola infection [75].

#### 5.4.3. Macroscopic Findings

Non-purulent perivascular inflammation was observed in the lungs of a male Eastern quoll (*Dasyurus viverrinus*) [75].

#### 5.4.4. Histopathological Findings

S. schenckii infection commonly presents a mixed suppurative and granulomatous inflammatory reaction in the dermis and subcutaneous tissue, mostly associated with microabscess and fibrosis [90]. Cutaneous infections may, furthermore, evince hyperkeratosis, parakeratosis, and pseudoepitheliomatous hyperplasia [73,90,93,97]. Granulomas in sporotrichosis usually contain necrotic debris, and are encompassed by plasma cells, caseous material, giant cells, lymphocytes, plasmocytes, and fibroblasts, as well as S. schenckii yeast cells [73,90]. Polymorphonuclear cells infiltrate the periphery of these granulomatous lesions. The aggregation of macrophages and hemorrhages with emphysema of the alveoli was observed in the lung sections of quolls (*Dasyurus sp.*) [75]. The skin sample from the quolls showed a mild-to-moderate nodular pyoderma with focal erosions, ulcers, purulent-necrotizing, and sometimes pyogranulomatous inflammation of the subcutis, hair follicles, and subcutaneous fatty tissue, and dilatations of the sweat glands [75].

## 6. Conclusions

Mycotic parasites are major plant and insect pathogens, but they are also important as etiologies of disease in vertebrates, causing a wide variety of infections in humans and animals [98,99,100]. Throughout the years, wildlife have been a major source of infectious diseases transmissible to humans. Wildlife act as a reservoir, which is a major public health problem [7]. The One Health initiative is a global plan for advancing collaborations in all aspects of health care for humans, animals, and the environment [101]. Despite the One Health approach towards the diagnosis of bacterial and viral zoonotic infections from wildlife, little effort is put into mycotic zoonotic infections [7,102,103]. In conclusion, mycoses of wildlife are of public health importance; therefore, veterinarians, human medics, public health specialists, zookeepers, and wild pet owners should be better informed about the etiology, epidemiology, diagnosis, and pathology of mycotic diseases of wildlife.

## Data Availability

Not applicable.
